# Research Progresses of Garnet-Type Solid Electrolytes for Developing All-Solid-State Li Batteries

**DOI:** 10.3389/fchem.2020.00468

**Published:** 2020-06-24

**Authors:** Abin Kim, Seungjun Woo, Minseok Kang, Heetaek Park, Byoungwoo Kang

**Affiliations:** Department of Materials Science and Engineering, Pohang University of Science and Technology (POSTECH), Gyeongbuk, South Korea

**Keywords:** all-solid-state-batteries, solid electrolyte, garnet-type electrolyte, Li ion batteries, beyond Li-ion batteries

## Abstract

All-Solid-State Batteries (ASSBs) that use oxide-based solid electrolytes (SEs) have been considered as a promising energy-storage platform to meet an increasing demand for Li-ion batteries (LIBs) with improved energy density and superior safety. However, high interfacial resistance between particles in the composite electrode and between electrodes and the use of Li metal in the ASBS hinder their practical utilization. Here, we review recent research progress on oxide-based SEs for the ASSBs with respect to the use of Li metal. We especially focus on research progress on garnet-type solid electrolytes (Li_7_La_3_Zr_2_O_12_) because they have high ionic conductivity, good chemical stability with Li metal, and a wide electrochemical potential window. This review will also discuss Li dendritic behavior in the oxide-based SEs and its relationship with critical current density (CCD). We close with remarks on prospects of ASSB.

## Introduction

Li ion batteries (LIBs) that use organic liquid electrolyte were commercialized in 1991 and have been developed to realize higher energy density and higher power density than other battery types. Applications include small electronic devices, and these are being extended to medium and large energy-storage areas such as electric vehicles (EVs) and the energy storage system (ESS). The EV market is expected to increase rapidly from about $ 22 billion in 2018 to about $ 118 billion in 2025 (Walia, 2019). To realize medium and large energy-storage devices, the energy density and safety of LIBs must be increased, and their cost must be decreased.

Many efforts have focused on the development of all-solid-state LIBs (ASSBs), which use a solid electrolyte and are therefore safer than conventional LIBs, which use a liquid electrolyte in a flammable solvent. Solid electrolyte (SE)s of ASSB are divided into polymer and ceramic ones, and many research studies have recently been performed on sulfide-based and oxide-based SEs (Oudenhoven et al., 2011; Kim et al., 2015; Zheng et al., 2018; Samson et al., 2019). In this paper, we review the garnet-type SEs in the oxide-based electrolyte and ASSB with them. The ASSBs with the oxide-based SEs have several advantages over conventional LIBs. Firstly, The ASSBs can increase achievable energy density because the oxide-based SEs can have much higher electrochemical potential window ≥5.0 V than the liquid electrolytes. Therefore, ASSBs can use cathode materials that have much higher redox voltage than the electrochemical voltage window of liquid electrolytes, which is typically <4.5 V (vs. Li). Furthermore, ASSBs can use a high capacity Li metal instead of the graphite anode, resulting in further increase in the energy density (Monroe and Newman, 2005; Thompson et al., 2017). This is because the oxide-based SEs can suppress the Li dendritic growth due to their high Young's modulus and its unity transfer number. Secondly, the ASSBs can achieve high volumetric energy density. When packaging cells for medium and large batteries, the cells with the liquid electrolytes must be assembled into a module and a pack after each cell is completely sealed and isolated because the liquid electrolytes are inter-mixed between cells. In contrast, the ASSBs can be directly stacked in sequence without isolating each cell because the oxide-based SEs are not inter-mixed. For this reason, it is expected that the volume of the ASSB can be reduced to 1/5, which leads to the improvement of the energy density per volume (Thangadurai et al., 2014; Janek and Zeier, 2016). Thirdly, the ASSBs are much safer than cells that use liquid electrolytes because the SEs are not flammable, whereas liquid electrolytes can act as a fuel during thermal runaway (Manthiram et al., 2017). Considering that the safety of large-scale battery systems such as electrical vehicles is the most important factor, superior safety of ASSBs will be remarkable advantage.

ASSBs can use ceramic solid electrolytes (SEs) (Oudenhoven et al., 2011; Kim et al., 2015; Zheng et al., 2018; Samson et al., 2019). Ceramic solid electrolytes can be mainly divided into sulfides and oxides. In the case of sulfide-based SEs, such as Li_10_GeP_2_S_12_ (LGPS), the ionic conductivity is high like 10^−2^ S/cm, which is as high as a liquid electrolyte can. Furthermore, the sulfide-based solid electrolytes can be easily integrated to the ASSB because they easily construct the ASSB even with a cold-press process (Kamaya et al., 2011). Toyota announced the prototype cell of a LiCoO_2_ cathode, sulfide solid electrolyte, and anode as Li_4_Ti_5_O_12_ in 2010, and they have then since focused on researching and developing the sulfide-based electrolytes and their solid-state cell for practical applications (Advanced Industrial Science and Technology, 2010). Even though the sulfide-based solid electrolytes for ASSB can address several issues, they still cause several problems. Firstly, they can react with oxygen and moisture in the air and then generate a deadly toxic hydrogen sulfide (H_2_S). Secondly, they can easily make detrimental interfacial reactions on the oxide-based active materials in composite electrode, leading to the formation of an insulating interfacial product that can increase the polarization and reduce electrochemical activity. These problems need additional process and cost for the sealing to avoid the contact with the air and the interfacial treatments. Thirdly, the sulfide-based electrolytes are difficult to further increase achievable energy density in spite of improved safety because they cannot use high capacity Li metal as an anode due to severe chemical/electrochemical reaction with Li metal, and they cannot use high voltage cathode materials due to their limited electrochemical potential window (Kamaya et al., 2011; Janek and Zeier, 2016; Kerman et al., 2017).

Even though the oxide-based SEs have lower ionic conductivity at the grain boundary and higher sintering temperature than sulfide-based electrolytes and have a difficulty with the integration of active materials in ASSBs, they have excellent chemical stability against various active materials and against high-capacity Li metal. As a result, ASSBs with oxide-based SEs have potential for use as next-generation batteries.

Among oxide-based SEs, we focus on the garnet-type ones that are represented by nominal composition of Li_7_La_3_Zr_2_O_12_ (LLZO) because they have high ion conductivity (10^−3^-10 ^−4^ S/cm), especially high grain boundary conductivity (Murugan et al., 2007). Garnet-type SEs are chemically stable against Li metal compared to Nasicon-type oxide-based SEs such as LATP(Li_1.7_Al_0.3_Ti_1.7_(PO_4_)_3_ and LAGP(Li_1.5_Al_0.5_Ge_1.5_(PO_4_)_3_). For example, LAGP has comparable ionic conductivity to LLZO SEs but shows mechanical/thermal failures as a result of the chemical reaction with Li metal (Chung and Kang, 2017). As a result, garnet-type LLZO SEs have been considered to be the most suitable SE for ASSBs because a solid-state cell with LLZO SEs can use a Li metal as an anode. They thus achieved higher energy density than existing ASSBs. In this short review, we will discuss the progress of the researches to improve ion conductivity in garnet-type SEs, studies on the interfacial reactions on the anode interface between Li metal and SEs, and studies on the cathode interface with SE and fabrication processes to realize ASSBs that use LLZO SEs.

## Progress in Ionic Conductivity of LLZO Garnet-Type SEs

Increase in the ionic conductivity of SEs is one of the priorities at the beginning of the development of the ASSB because poor ionic conductivity of SE in ASSB compared to liquid electrolytes causes severe polarization of the voltage during cycling and thereby reduces the energy efficiency of the ASSB (Liu C. et al., 2016). In Table 1, the activation energy and conductivity according to the chemical composition of LLZO were investigated.

**Table 1:** Ion conductivity of garnet-type solid electrolytes.

**References**	**Chemical formula**	**σ_Li+_** **at RT [mS/cm]**	**E_**a**_[eV]**	**Synthesis condition (solid-state reaction)**
Murugan et al. (2007)	Li_7_La_3_Zr_2_O_12_	0.30	0.32	1,230°C, 36 h
Ohta et al. (2012)	Li_6.75_La_3_Zr_1.75_Nb_0.25_O_12_	0.80	0.31	1,200°C, 36 h
Yao et al. (2015)	Li_7.06_La_3_Y_0.06_Zr_1.94_O_12_	0.81	0.26	1,200°C, 16 h
Thangadurai et al. (2014)	Li_6.6_La_3_Zr_1.6_Sb_0.4_O_12_	0.77	0.34	1,100°C, 24 h
Alexander et al. (2018)	Li_6.28_Al_0.24_La_3_Zr_2_O_12_	0.44 (30°C)	0.37	1,200°C, 12 h
Umeshbabu et al. (2019)	Li_5.9_Al_0.2_La_3_Zr_1.75_W_0.25_O_12_	0.49	0.35	1,150°C, 12 h
Lu et al. (2018)	Li_6.25_Ga_0.25_La_3_Zr_2_O_12_	1.46	0.25	1,100°C, 24 h
Wu et al. (2017)	Li_6.20_Ga_0.30_La_2.95_Rb_0.05_Zr_2_O_12_	1.62	0.26	1,100°C, 4 h

**Figure 1:**
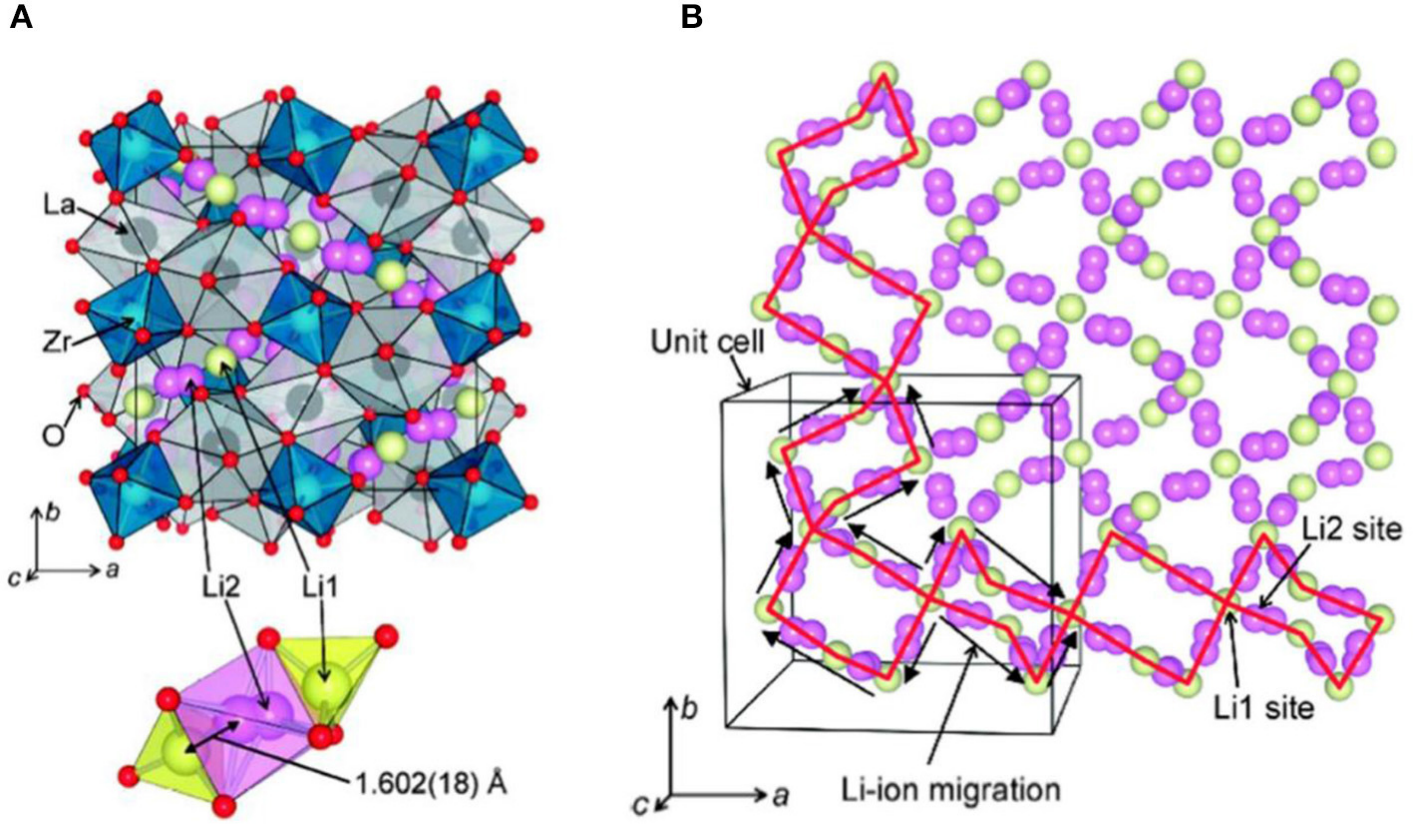
**(A)** LLZO crystal structure and **(B)** Li ion conduction channel (Li1−24 d, Li2–96 h) [Adapted from Awaka et al. (2011) with permission from Chemical Society Japan].

Murugan et al. (2007) first reported a garnet-type LLZO SE, which has high ion conductivity (10^−3^-10^−4^ S/cm) in grains and at grain boundaries. In LLZO structure (Figure 1A), ZrO_6_ octahedra, and LaO_8_ dodecahedra are connected to form a structure, and Li ions (Li^+^) and Li vacancies *V*_Li_ are located in the invasive position of the tetrahedral sites and octahedral sites. One Li^+^ is located at the tetrahedral 24 d position, and one is located at the octahedral 96 d position. As a result, the conduction channel (Figure 1B) for Li^+^ in LLZO structure is 24 d → 96 h → 24 d (Wagner et al., 2016).

The LLZO has two polymorphs: a cubic phase (*c*-LLZO) and a tetragonal phase (*t*-LLZO). At room temperature (RT), the cubic phase has 100 times higher ionic conductivity (~10^−4^ S/cm) than the tetrahedral phase (~10^−6^ S/cm). The high conductivity of the *c*-LLZO originates from the uniform movement of Li^+^ ions in the *x, y*, and *z* directions, whereas *t*-LLZO has low conductivity because Li^+^ ions move only in the *x* and *y* directions (Chen et al., 2018). Different distributions of Li^+^ in the two phases also further affect the Li ionic conductivity. In the cubic phase, the Li sub lattice is disordered (partial occupancies in Li symmetry sites), whereas in the tetragonal phase it is ordered (either full or empty occupancies in Li sites). When all the Li sites are empty or full, *t*-LLZO can have a lower electrostatic energy than *c*-LLZO because *t*-LLZO can reduce the Coulombic repulsion among Li^+^ ions (Bernstein et al., 2012). As a result, *t*-LLZO is thermodynamically more stable than *c*-LLZO at RT.

At the beginning, most research studies were devoted to stabilizing *c*-LLZO over *t*-LLZO at RT to achieve high ionic conductivity. Doping can stabilize the cubic phase. For example, if Ta^5+^ and Nb^5+^ ions are doped into Zr^4+^ sites, or Al^3+^ and Ga^3+^ are doped into Li^+^ sites, the number of *V*_Li_ is changed, and the disorder can thus be developed at the Li and *V*_Li_ sites. The disorder of Li and vacancies increases the configuration entropy at RT, and the Gibbs free energy of the *c*-LLZO thus decreases, resulting in the stabilization of *c*-LLZO over *t*-LLZO at RT (Thangadurai and Weppner, 2005; Allen et al., 2012; Huang et al., 2012; Wolfenstine et al., 2012; Ramakumar et al., 2013; Rangasamy et al., 2013; Thompson et al., 2014; Xia et al., 2016; Buannic et al., 2017; Song et al., 2019a).

**Figure 2:**
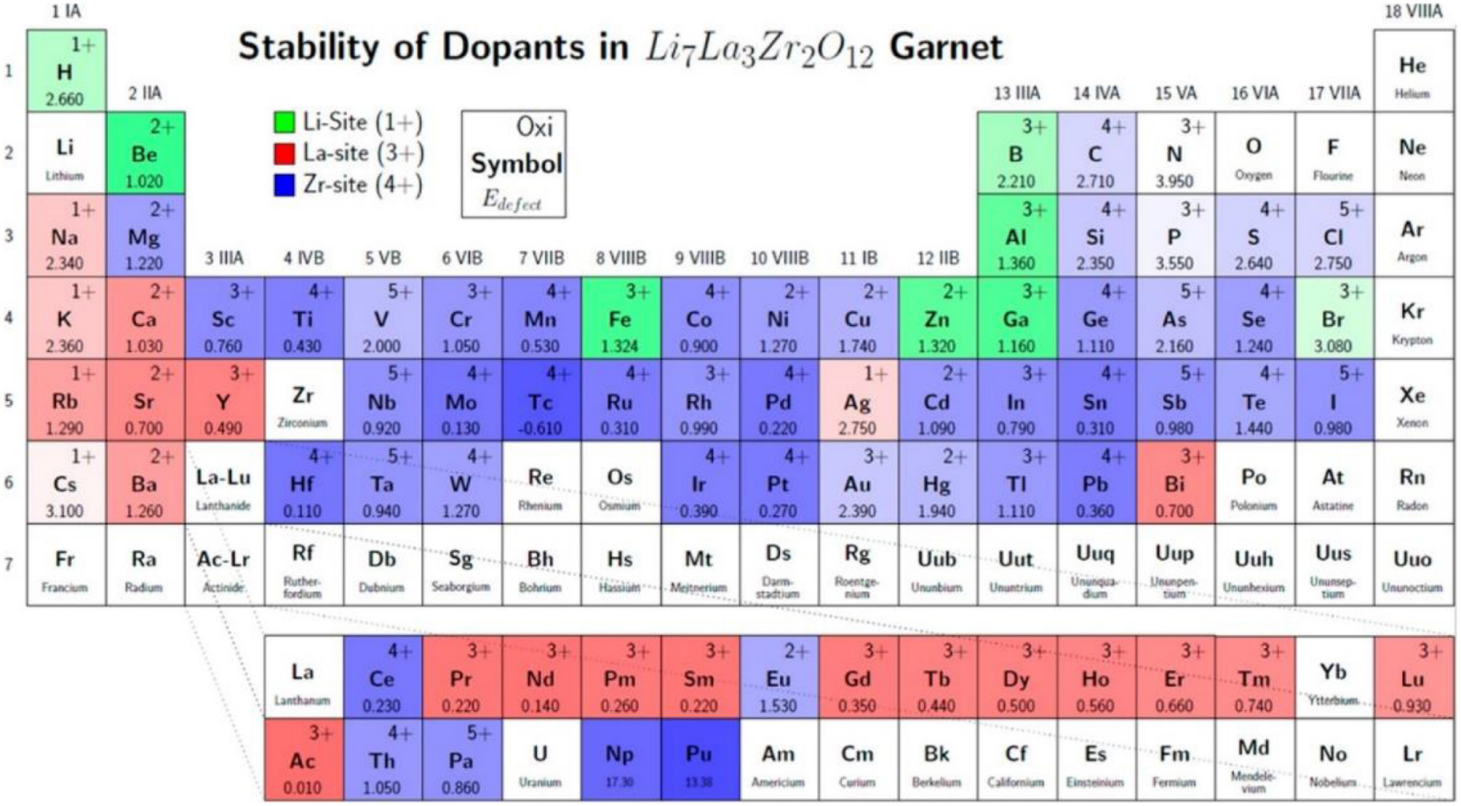
Site and oxidation state preference of doping elements in LLZO structure studied by DFT calculations. The color shows the most stable cation site (green: Li site, red: La site, blue: Zr site). Defect energy decreases as darkness increases [Reproduced from Miara et al. (2015) with permission from American Chemical Society].

To increase the ionic conductivity of garnet-type SEs by doping, the sites and the oxidation state preferred by each dopant have been identified using first-principle calculations that exploit density-functional theory (DFT) (Figure 2). This doping strategy can further increase the Li ionic conductivity in the LLZO SEs (Miara et al., 2015).

The other way to increase ionic conductivity in LLZO SEs is through an increase in the pellet density by using sintering agents, which can decrease the sintering temperature and the number of grain boundaries. For example, Li_3_BO_3_ (LBO) as a sintering agent has been considered for LLZO SEs to increase the conductivity of LLZO at a lower temperature than the typical sintering temperature of LLZO SEs, 1,100–1,250°C (Shin et al., 2016). When LLZO SEs are sintered below the sintering temperature, they develop high porosity or a tetrahedral phase, and they thus have low Li ionic conductivity partly due to low pellet density. However, when the LLZO is sintered with LBO above the melting point of LBO (~850°C) but below the sintering temperature of LLZO, a liquid phase sintering occurs, and the LLZO can therefore achieve high pellet density leading to high ionic conductivity (Shin et al., 2016). Low-temperature sintering of LLZO helps to suppress evaporation of Li and to reduce the chemical reactivity between LLZO SEs and active materials in composite electrodes when ASSBs are fabricated by using a co-sintering process, which is a feasible integration process.

High ionic conductivity in LLZO SEs is also obtained by a special sintering process such as Field Assisted Sintering Technology (FAST) (Zhang et al., 2017), which uses electric field in sintering. During the FAST process, LLZO is sintered in a short time due to additional electric field and develops high pellet density (~99.8%). As a result, LLZO SEs can achieve high Li^+^ conductivity (~1.01 × 10^−3^ S/cm). The FAST process can be useful for fabricating ASSB because the short sintering time does not allow diffusion between SEs and active materials.

Recently, processes such as tape-casting are being scaled up in LLZO SEs because the tape-casting process is well-developed in the SOFC (Solid Oxide Fuel Cell) field and can be done by a roll-to-roll process. Long, dense LLZO SE thin film (40–50 μm) has been obtained using the tape-casting; this success indicates that the scale-up of LLZO SEs for ASSBs can be feasible (Fu et al., 2016; Xie et al., 2019). Given that the SEs in ASSB are not active component, a thin dense SE is desirable. Thin dense SEs also have the merit that their thickness can be reduced, and so the ionic resistance decreases even though the ionic conductivity is not increased (Fu et al., 2016; Xie et al., 2019). The total resistance of SEs is inversely proportional to their thickness, so thin LLZO SE can substantially reduce the large polarization of voltage that a thick SE can cause (Fu et al., 2016; Xie et al., 2019), leading to the improvement of energy efficiency.

## LI Metal Anode With LLZO SEs

Garnet-type LLZO SEs generally have good chemical and electrochemical stability with Li metal, which is considered to be the best anode material due to its low redox potential and high capacity (Thangadurai et al., 2003; Murugan et al., 2007; Han et al., 2017; Hofstetter et al., 2018). However, challenges, such as poor wetting behavior with Li metal and Li dendritic growth inside LLZO SEs, must be overcome before ASSB with LLZO SEs are viable in practical applications.

### Li Metal/Garnet-Type Electrolyte Interface: Poor Interfacial Wetting Behavior

**Figure 3:**
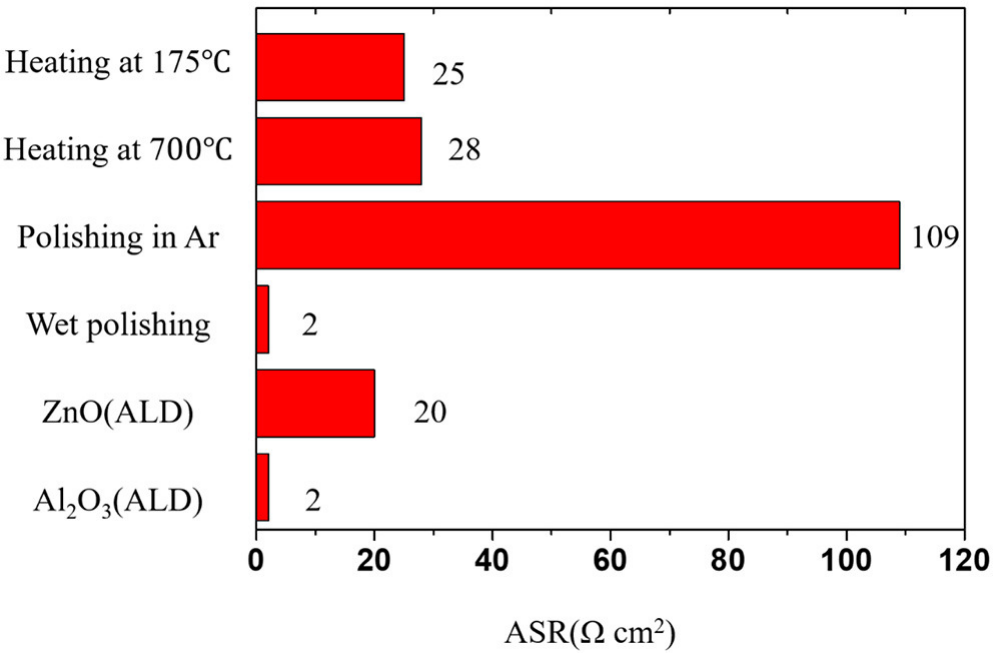
The known strategies to reduce area-specific resistance (ASR) between Li metal and LLZO SEs [Drawn from the data of Wang et al. (2017), Fu et al. (2017a), Han et al. (2017), Luo et al. (2017)].

Garnet-type LLZO SEs are not wetted well by Li metal, and so the interface between an SE an Li metal does not form intimate physical contact (Kotobuki et al., 2010; Buschmann et al., 2011; Fu et al., 2017a). Poor physical contact causes high interfacial resistance (~10^3^ Ω) and inhomogeneous current distribution at the interface leading to the formation of hot spots. As a result, inhomogeneous current distribution at the interface can cause dendritic growth of Li metal in SEs during cycling. The poor wetting behavior with Li metal in LLZO SEs is partly a result of the presence of contaminants such as Li_2_CO_3_ and LiOH on the surface. The contaminants are insulators to both ionic and electronic movement (Cheng et al., 2015; Xia et al., 2016; Sharafi et al., 2017b,c). To achieve high energy density in ASSB based on LLZO SE and Li metal, the wetting behavior with Li metal must be improved, and the interfacial resistance must be decreased. The area-specific resistance (ASR) for total resistance of a commercial Li-ion battery have been reported ~22 Ω cm^2^. The ASR for a practical ASSB with LLZO should thus be comparable or lower than the ASR of commercial cell (Hitz et al., 2019). Accordingly, many studies have been conducted to improve the interfacial reactions between the garnet-type LLZO SEs and the Li metal (Figure 3).

A simple approach to improve poor wetting behavior is to remove the contaminants on the surface through a polishing process or heat treatment. For example, a polishing process can improve physical contact between LLZO SEs and Li metal by removing the insulating Li_2_CO_3_ surface layer that forms by reaction with the air. When the contamination layers (Li_2_CO_3_, LiOH) are removed by wet polishing with glycol-based diamond paste extender and polishing fluid, the interfacial resistance of LLZO SE is substantially reduced to 2 Ω·cm^2^ (Sharafi et al., 2017b). Addition of 2 wt% LiF during synthesis can suppress the formation of Li_2_CO_3_ on the surface because LiF on the surface can prevent the diffusion of moisture and CO_2_ from the air. Application of LiF to LLZO SEs can reduce the interface resistance between Li metal and LLZO SE from 1,260 to 345·Ω cm^2^ (Li et al., 2017). These results demonstrate that the poor wetting behavior at the interface of LLZO SE with Li metal may be partly attributable to surface contaminants. Even though these approaches can reduce the interfacial resistance, the effectiveness is limited because complete removal of the contaminant layer is not possible.

Several approaches to improve interfacial properties also have been developed by using a coating layer on the surface. One approach is to deposit a material that wets Li metal well (e.g., Al_2_O_3_, ZnO, Al) on the surface of LLZO SEs by using Atomic Layer Deposition (ALD) (Fu et al., 2017a; Han et al., 2017; Wang et al., 2017) or thermal deposition (Tsai et al., 2016; Luo et al., 2017). The deposited materials on the surface significantly improve the wettability of LLZO SEs with Li metal, and the interface resistance is thus substantially reduced. ALD deposition of Al_2_O_3_ on the surface substantially reduces the ASR of LLZO from 1,710 to 1 Ω cm^2^ at RT due to formation of Li-Al-O products that can form homogenous physical contact or can suppress the formation of Li_2_CO_3_ on the surface (Han et al., 2017). The ALD deposition of ZnO on the surface also can easily form a Li/Zn alloy by using molten Li to achieve homogeneous physical contact and reduce the interface resistance to as low as ~20 Ω cm^2^ (Wang et al., 2017).

Thermal deposition of Ge can decrease the garnet/Li-metal interfacial resistance from 900 to 15 Ω cm^2^ due to an alloying reaction between the Li metal and the Ge to form homogeneous interface contacts (Luo et al., 2017). These results demonstrate that methods to achieve homogeneous physical contact between LLZO and Li metal can effectively reduce the interfacial resistance.

### The Dendritic Li Metal Growth Inside LLZO SEs

Garnet-type LLZO SEs were believed to be able to prevent Li dendrite growth because of their high Li transference number and because the shear modulus of Li-garnets is an order of magnitude higher than that of Li metal (Brissot et al., 1999; Monroe and Newman, 2005; Janek and Zeier, 2016; Yu et al., 2016). However, dendritic growth of Li metal or Li metal island has been observed inside LLZO SEs (Ren et al., 2015; Tsai et al., 2016; Kerman et al., 2017; Shen et al., 2018; Tian et al., 2018, 2019).

Li dendrites can form along pre-existing defects such as grain boundaries and voids in SEs (Cheng et al., 2015; Kerman et al., 2017; Sharafi et al., 2017a) because defects and cracks can permit much higher electron diffusion than perfect grains, and this allows Li dendrites to propagate.

Li dendrites can also grow in SEs that do not have any defects or voids or that are single crystals without the grain boundary. For example, Li dendrites can grow even in very dense LLZO SE (relative density >97%) (Tsai et al., 2016; Cheng et al., 2017; Yonemoto et al., 2017) that has a negligible number of voids. Single-crystal LLZO SEs can still permit internal growth of Li metal dendrites even though the single crystal does not have any grain boundary, which was considered as an important factor that permits Li dendrites (Porz et al., 2017).

The formation of Li dendrites also strongly depends on the applied current in the LLZO SE. Inhomogeneous current distribution caused by the insufficient physical contact between Li metal and SEs may lead to the formation of Li dendrites (Cheng et al., 2015; Sharafi et al., 2016, 2017b; Tsai et al., 2016). Even when interfacial resistance at the interface is significantly reduced by surface modifications, Li dendrites still form even at a very low current density (<0.9–1 mA·cm^−2^) (Han et al., 2019). To describe the dependence of Li dendrite behavior on the applied current density, the concept of critical current density (CCD) was developed. The critical current density is defined by the applied current density that causes the short-circuit of SEs. Low CCD in the SEs means that SEs have dendritic Li growth at a low current density. It indicates the failure of the cell caused by Li dendrites, and it is thus a useful parameter to determine whether the SE in an ASSB can be used in practical applications. When an SE has perfect properties but low CCD, the ASSB that uses the SE cannot be charged or discharged quickly. Many research efforts have focused on understanding the low CCD of LLZO SEs, and on how to increase it.

The relatively high electronic conductivity of the LLZO SE (10^−8^-10^−7^ S cm^−1^) may be responsible for low CCD of the LLZO SE (Aguesse et al., 2017; Tian et al., 2018; Han et al., 2019; Song et al., 2019a). Relatively high electronic conductivity can lead to deposition of Li metal at or near grain boundaries in the LLZO SE (Song et al., 2019b). To reduce the electronic conductivity at the grain boundary in LLZO SEs, a thin layer of LiAlO_2_ as an electronic insulator can be coated on the surface of the grain; this process increases the CCD from 0.45 to 0.75 mA·cm^−2^ (Song et al., 2019b).

A new model of the growth of Li dendrites in LLZO SE suggests that a high external voltage applied across an LLZO SE can break the energy barrier of the grain and facilitate transfer of electrons (Song et al., 2019a). To avoid this breakdown behavior, the energy barrier for the electron transfer at grain boundaries must be increased. Low CCD in SEs does not allow fast charging or discharging, which needs high current density, and the CCD of LLZO SEs must therefore be significantly increased before they can have practical applications.

**Figure 4:**
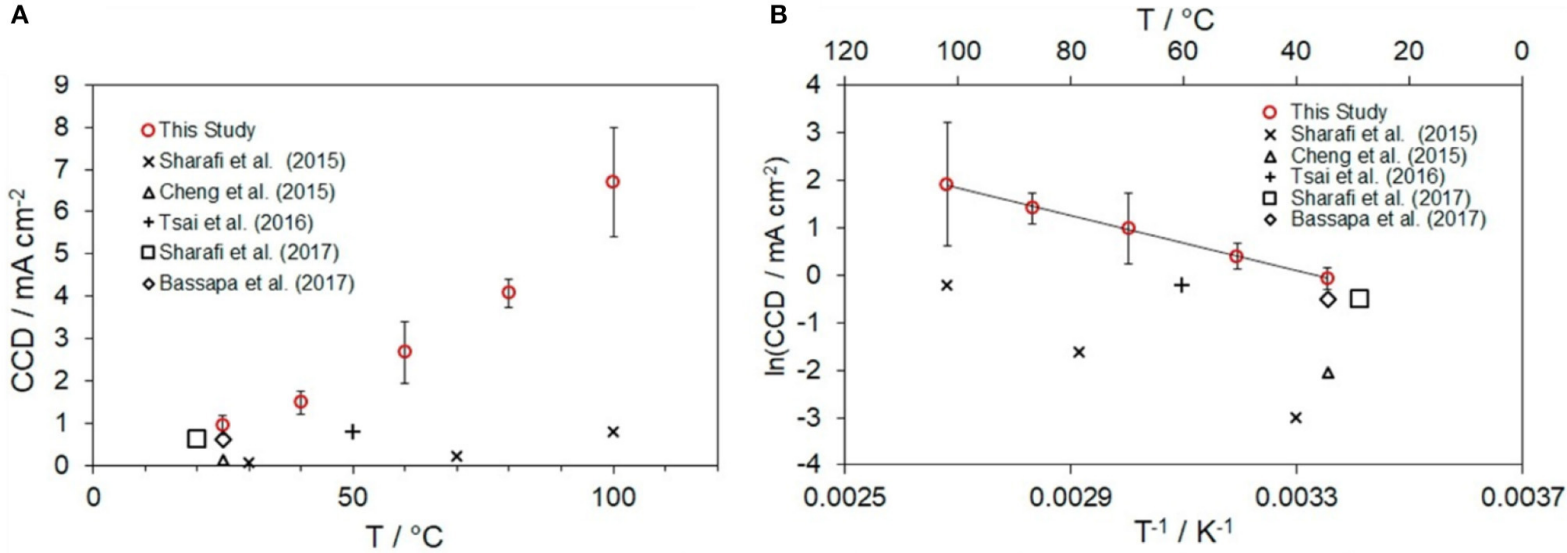
**(A)** Critical current density (CCD) of LLZO SEs as a function of temperature and **(B)** Arrhenius plot of CCD (Cheng et al., 2015; Sharafi et al., 2016, 2017a; Tsai et al., 2016; Basappa et al., 2017; Wang M. et al., 2019 [Adapted from Wang M. et al. (2019) with permission from Elsevier].

Given that ASSBs can operate at wide range of temperatures, the effect of the temperature on CCD should be understood. CCD increases exponentially with temperature (Figure 4) (Sharafi et al., 2016; Wang M. et al., 2019). Even though the same LLZO SE is used, the CCD can be ~1 mA·cm^−2^ at RT but ~7 mA·cm^−2^ at 100°C before the device short-circuits (Wang M. et al., 2019). This relationship between temperature, and CCD is not well-understood. One of reason may be the difference between the flux of Li^+^ ions toward the interface and the flux of Li metal away from it; at high CCD, the flux of Li^+^ ions toward the interface may exceed the flux of Li metal away from the interface, and so “hot-spots” can form; they sharply increase the local overpotential at the interface (Wang M. et al., 2019). To use a high-capacity Li metal as an anode in an ASSB that uses the LLZO SE, the low CCD of the LLZO SE must be increased. This progress requires improved understanding of the properties of the CCD in SEs. It should be noted that the CCD depends on not only SEs' properties such as electronic conductivity and ionic conductivity but also the preparation parameters and surface roughness of SE.

Recently, Yonemoto et al. claimed additional problem in using Li metal in ASSB (Yonemoto et al., 2017). During electrochemical cycles, repeated deposition/strip of Li metal affects mechanical stability of Li metal via the formation of voids, and thereby the polarization increases. Increasing the number of voids as a function of cycles increases the polarization, which easily leads to the Li dendritic growth, and short-circuits can thus happen easily as the number of cycles increases.

## The Ways to Integrate Cathode With LLZO SE

To fabricate ASSBs with oxide-based SEs, the interface between SEs and active particles in the composite cathode and the interrace between the composite cathode and SE should also be improved because cell resistance mainly originates from the interfaces in cathode rather than from the SE (Sakuda et al., 2008). The interface between the composite cathode and oxide-based SE is not well-constructed, and the process for constructing the interface is not well-developed. In contrast, in the sulfide-based SEs, the interfaces between cathode and sulfide-based SEs as well as between particles in a composite electrode are well-constructed by simply applying pressure because the sulfide-based SEs are mechanically soft (Sakuda et al., 2013). Oxide-based SEs have hard mechanical characteristics, and much of the research on oxide-based SEs has therefore been focused on reducing interfacial resistances between active particles and SEs in a composite electrode, and between a composite electrode and dense SEs, and on developing a simple process to build these interfaces and ASSBs. ASSB are summarized in Table 2 by classifying the manufacturing method.

**Table 2:** LLZO-based ASSB with Li layered transition metal oxides as active cathode materials.

**References**	**Experimental techniques**	**Cathode composition**	**Active** **material loading (mg/cm^2^)**	**Current density uA/cm^**2**^ (C-rate)**	**Cut-off Voltage(V)**	**Discharge Capacity (mAh/g)^**b**^**	**Cycle No**.	**Operating T(^**°**^C)**
Kotobuki et al. (2010)	Sol-gel thin film	LiCoO_2_ sol-gel film	N/A	2	2.5–4.3	0.015	3	RT
Ohta et al. (2011)	PLD	LiCoO_2_	N/A	3.5	2.5–4.2	125		25
Ohta et al. (2012)			0.255^a^	3.5 (0.1C)	2.5–4.2	129	100	25
Kato et al. (2014)			N/A	1	3.2–4.2	80	25	RT
Ohta et al. (2013)	Co-sintering	LiCoO_2_ with LBO	1.7^a^	10 (0.05C)	3.0–4.05	85	5	25
Ohta et al. (2014)			0.73^a^	1 (0.01C)	3.0–4.2	78	1	25
Park et al. (2016)		LiCoO_2_ with LBO + LLZO	N/A	N/A (0.2C)	2.5–4.4	67	10	50
Liu T. et al. (2016)		LiCoO_2_ with LBO + In_2_Sn_2_O_5_	1.2	5 (0.025)	2.8–4.3	101	1	RT
Han et al. (2018)		LiCoO_2_ @ Li_2_CO_3_ with	1	5.7 (0.05C)	3.0–4.05	106	40	100
		LCBO + LLZO @ Li_2_CO_3_				94	100	25
Liu et al. (2018)		Li[Ni_0.5_Co_0.2_Mn_0.3_]O_2_ with LBO + ITO	1	5 (C/30) ^a^	3.0–4.6	123	5	80
Wang D. et al. (2019)		Li[Ni_0.6_Mn_0.2_Co_0.2_]O_2_	1.5–2.0	8.6–11.5^a^	3.0–4.2	106 (small)	30	RT
		with LBO + LLZO		(0.05C)		65 (large)		
Ren et al. (2017)	Duplex structure	LiCoO_2_	2.9	6.4 (0.016C)	3–4.2	18	10	80
Zhang et al. (2017)	Polymer electrolyte	LiCoO_2_ with PEO	N/A	N/A (0.1C)	2.7–4.2	136	5	60

a Cannot confirm from the paper but can infer through calculation

b *Result of 1st cycle*.

### Pulsed Laser Deposition (PLD) Process to Construct Interface Between SE and Cathode

A thin film deposited by pulsed laser deposition (PLD) can provide a successful interface between the cathode and LLZO SEs for an operating ASSB (Ohta et al., 2012; Kato et al., 2014). Deposition of thin film by PLD can yield a well-constructed interface with low interfacial resistance. In a thin-film ASSB, the cathode/electrolyte interfacial resistance was 170 Ω·cm^−2^, which is as low as a liquid electrolyte. It should be noted that the thin-film ASSB does not have the interface between active particles and SEs because it uses the deposition of the cathode.

**Figure 5:**
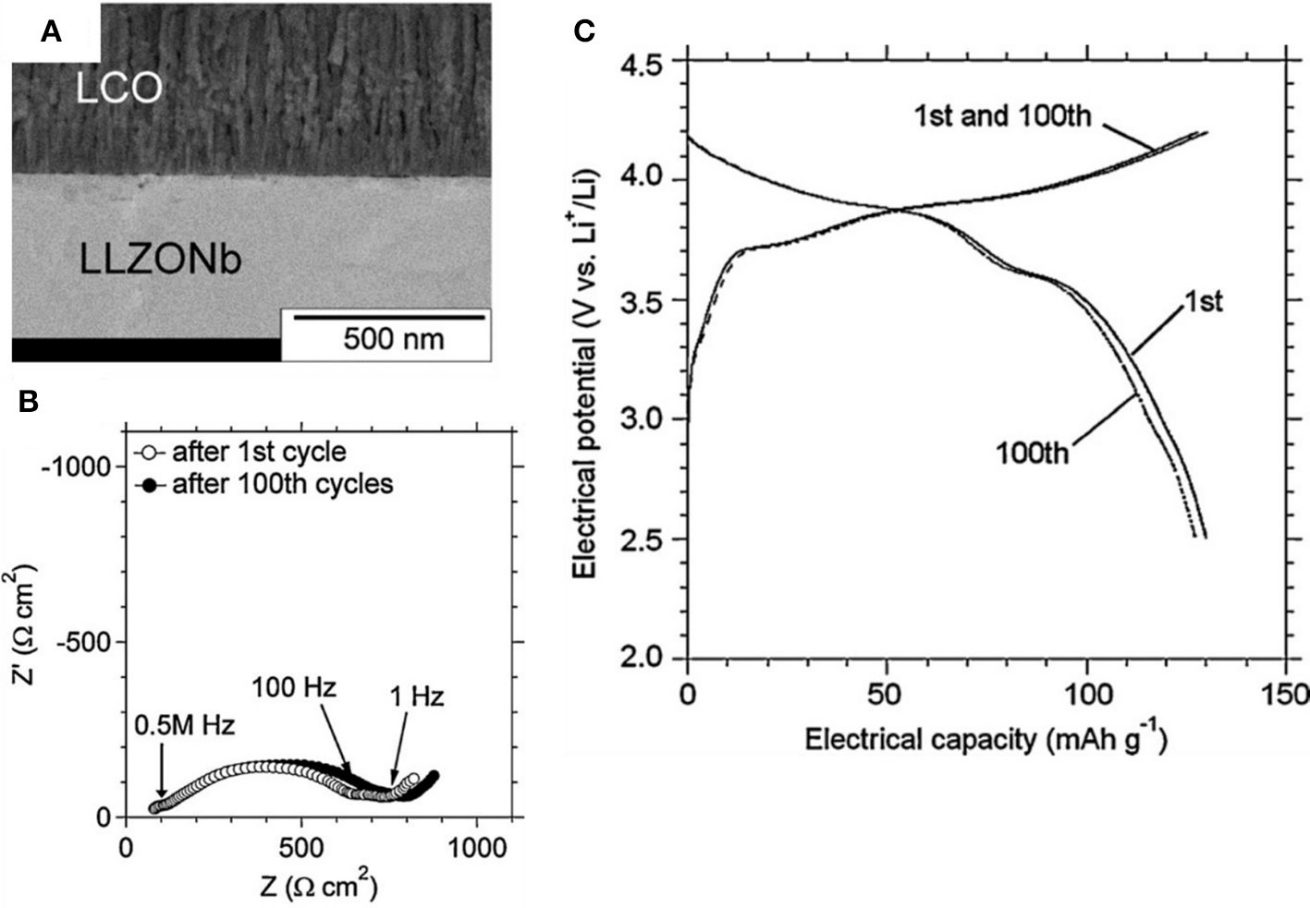
**(A)** FE-SEM of cross-sectional image **(B)** Nyquist plot (0.1 Hz to 1 MHz) **(C)** Charge–discharge curves and after 100 cycle for LiCoO_2_/Li_6.75_La_3_Zr_1.75_Nb_0.25_O_12_/Li thin film battery [Adapted from Ohta et al. (2012) with permission from Elsevier].

The thin-film ASSB can be operated with negligible capacity fading for cycles because of its unique monolithic structure with a thin layer of cathodes (Figure 5) (Ohta et al., 2012). A thin-film battery has advantages of small size and small weight, and so it is useful to power small applications such as smart cards. However, a thin-film battery can contain only a small amount of active materials; its energy density is therefore low compared to existing Li-ion batteries or other secondary batteries. Thin-film ASSBs has low energy density (38 Wh/L) that is 10 times lower than currently developed LIB batteries (Moitzheim et al., 2019). Low energy density limits the application of thin-film ASSBs. Therefore, high-capacity ASSB should be developed by using bulk-type electrodes that have high loading of active materials. Use of these bulk-type electrodes in ASSBs requires development of a feasible fabrication process that can combine bulk-type electrodes with oxide-based SEs and Li metal but does not use thin-film technology.

### Fabrication Process of Cathode/Garnet-Type SE Interface: Co-sintering Process

Several processes can be used to fabricate an ASSB that has bulk-type electrodes. One such process uses co-sintering, which uses high temperatures to form a cathode/electrolyte interface that has low interfacial resistance. Applying high temperature to the mix of a composite cathode and LLZO SE can cause a chemical reaction via the inter-diffusion between the two components by thermal diffusion. If the elements in the cathode and the electrolyte react with each other, both components lose their original properties and cannot be used in an ASSB. For example, in co-sintering LCO and Al-doped LLZO at 700°C (Park et al., 2016), the Al is intended to stabilize the cubic phase of LLZO but also diffuses into LCO. As a result, *c*-LLZO is not stable and is transformed to *t*-LLZO leading to the decreases in the Li^+^ conductivity. This low conductivity severely increases the interfacial resistance and thereby degrades the overall performance of ASSB (Thompson et al., 2014). Also, Co, La, and Zr can inter-diffuse at the interface between LiCoO_2_ (LCO) and LLZO when high temperature is applied to make homogeneous contact at the interface (Kim et al., 2011; Kato et al., 2014; Vardar et al., 2018). Therefore, a fabrication process must be developed to build homogeneous contacts at the interface without decreasing the thermo-chemical stability of the cathode and electrolyte.

The thermal stability of the cathode/electrolyte interface can be quantified by determining whether secondary phases form in the mixture of active materials with LLZO SEs when it is annealed at high temperature. This method has been used to quantify the thermo-chemical stability of Li_6.73_La_3_Zr_1.73_Ta_0.23_O_12_ (LLZTO) on various cathode materials, such as LiCoO_2_ (LCO), Li(NiCoMn)_1/3_O_2_ (NCM) with a layered structure, LiMn_2_O_4_ (LMO) with a spinel structure, and LiFePO_4_ (LFP) with an olivine structure (Ren et al., 2016). The results of secondary phase formation indicate that LCO and NCM show higher thermo-chemical stability than LMO and LFP.

Heat treatment of LCO does not cause change or development of secondary phases, but heat treatment of LCO with LLZO SEs causes inter-diffusion behavior at the interface. These measurements show that the secondary phase such as the reaction product LaCoO_3_ form during heat treatment at 900°C. Other reports also suggest that reactions between the cathode materials and the LLZO SEs occur during heat treatment (Miara et al., 2015, 2016; Park et al., 2016; Vardar et al., 2018).

LLZO SEs need very high temperatures (>1,000°C) for sintering, and so the formation of dense composite electrode with LLZO SE by heat treatment needs temperature >1,000°C, which can cause chemical reactions. This reaction at the interface can be avoided by lowering the temperature that densifies the composite of the electrode with SEs.

Additives can decrease the sintering temperature during fabrication of a composite electrode with SEs. They reduce the temperature that is required to achieve intimate contact between LLZO SEs and active materials (Ohta et al., 2013; Han et al., 2018; Liu et al., 2018). Li_3_BO_3_ (LBO) has been suggested as an additive for the composite cathode with LLZO SE because LBO has a melting temperature of 700°C, which is much lower than the sintering temperature of LLZO SEs (>1,000°C).

Adding LBO to a composite of LCO with LLZO SE can lead to formation of a composite cathode that is intimate interfacial contact between the two components. However, the LBO has a low Li^+^ conductivity as 1.4 × 10^−9^S·cm^−1^(Jung et al., 2018), leading to induced high polarization. As a result, the overall ASSB performance is not significantly improved due to poor ionic conductivity of LBO. One way to solve this problem is to use a solid solution of Li_3_BO_3_-Li_2_CO_3_ (LBCO) as an additive to increase the ionic conductivity of the additive and decrease the fabrication temperature (Han et al., 2018).

**Figure 6:**
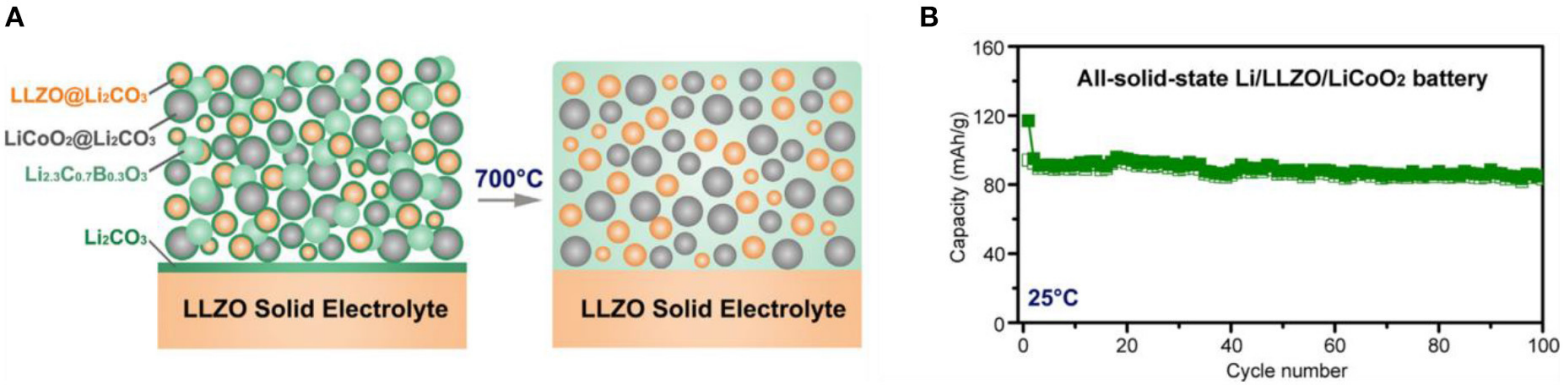
**(A)** Schematic diagram of Interphase-Engineered All-Ceramic Cathode/Electrolyte by using sintering agent LCBO **(B)** Cycling stability of the Li/LLZO/LCO cell using LCBO at 0.05C at 25°C [Adapted from Han et al. (2018) with permission from Cell Press].

LCBO has a similar melting point as the LBO and a relatively high Li^+^ conductivity of 3.4 × 10^−7^-1.2 × 10^−6^ S·cm^−1^ (Nagao et al., 2016). To form LBCO in the ASSB with LLZO SEs, heat treatment at low temperature can cause LBO to react with Li_2_CO_3_, which typically exists on the surface of the garnet-type SEs and LCO as a contaminant. As a result, fabrication of ASSB with LCO as an active material, LCBO as a sintering agent, and LLZO SE as an electrolyte can be simplified, and the performance of ASSB can be improved. This strategy yielded a cell that achieved reversibly for 100 cycles at 0.05C-rate at 25°C (Figure 6) (Han et al., 2018).

**Figure 7:**
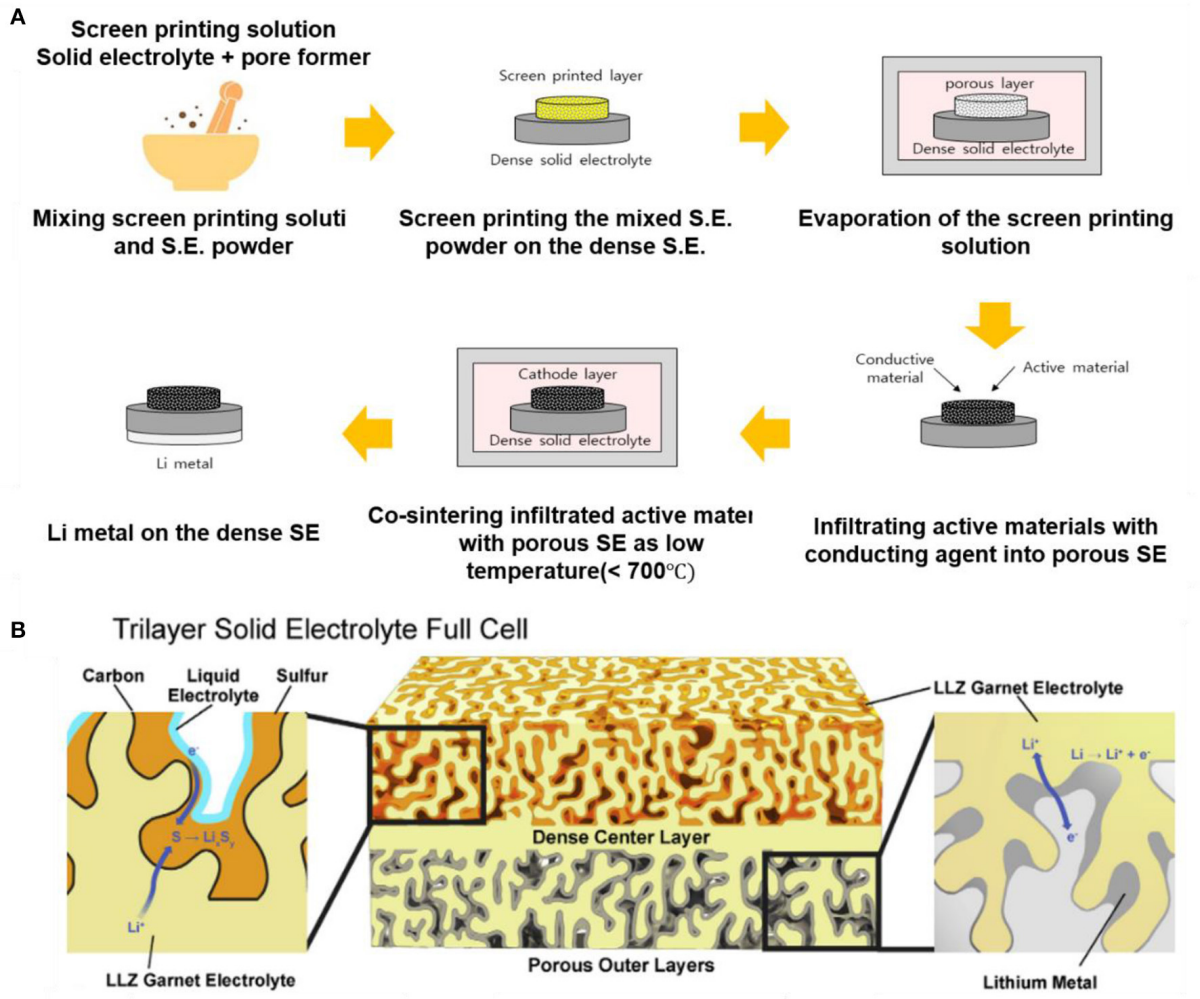
**(A)** Typical fabrication process of ASSB with porous/dense LLZO duplex structure. **(B)** Diagram of a tri-layer lithium garnet cell by using tape-casting process [Adapted from Hitz et al. (2019) with permission from Elsevier].

Fabrication of a duplex structure or use of a polymer electrolyte (Fu et al., 2017b; Ren et al., 2017; Zhang et al., 2017; Shen et al., 2019) can also suppress the reaction at the interface between cathode and LLZO during heat treatment. The duplex structure is composed of a porous SE layer, which can act as a cathode, on a dense SE layer as an electrolyte. This duplex structure achieves good connection between the porous SE and the dense SE; the interfacial resistance between SEs and cathode can be substantially decreased, and the Li^+^ conductivity inside the composite cathode can be increased. In the duplex structure, the active materials and conducting agent can be infiltrated into the porous SEs by using a slurry method or a screen-printing process. The duplex structure can also lower the temperature that is required to form a composite cathode because the porous/dense LLZO duplex structure by itself can be separately heated at >1,000°C before adding the active materials and conducting agent (Figure 7). As a result, the heat-treatment temperature for achieving intimate contacts between infiltrated active materials and SEs in the cathode part can be much lower than the sintering temperature of LLZO SEs. Recently, Wachsman's group showed the possibility of roll-to-roll mass production of LLZO SE by using tri-layer SE structure (porous|dense|porous SE), which is the same as the duplex structure, by using the tape-casting process (Figure 7). Using LLZO powder synthesized with a solid-state reaction, tape slurries were prepared by mixing with appropriate binders, plasticizers, and solvents. The porous-dense-porous LLZO tri-layer structure results in a low resistance, ~2–10 Ω·cm^−2^, which is much lower than the ASR of commercial full cell, 22 Ω·cm^−2^, mechanically robust structure capable of high-rate lithium cycling (Han et al., 2017).

The manufacturing process of the duplex structure is similar to that of solid oxide fuel cells (SOFC) and multi-layer ceramic capacitors (MLCC). As a result, it shares several processes such as tape-casting/screen printing, sintering, and stacking process. Therefore, the cost and scalability of the manufacturing process in the duplex structure in ASSBs can be estimated by using these technologies. According to recent review from Schnell et al., the cost of manufacturing ASSBs using LLZO SE is expected to cost 75–240 $ kW^−1^h^−1^ in making 100 million hybrid electric vehicle (HEV) cellsper a year (Schnell et al., 2019). In order to reach such a process cost, it is expected to develop innovative infiltration and electrode interfacial treatment technologies.

The ASSB in duplex structure or in tri-layer structure still needs to be improved because it needs a small amount of liquid electrolyte in the cathode or must be operated at a high temperature (80–120°C) to further improve electrochemical performance (Fu et al., 2017b; Ren et al., 2017; Shen et al., 2019). Moreover, a soft polymer electrolyte such as polyethylene oxide (PEO) has a relatively low Li^+^ conductivity ~10^−8^ S·cm^−1^ at room temperature (Croce et al., 1998); as a result, the ASSB with PEO electrolyte does not operate well at room temperature but does operate well at temperature >60°C, at which PEO is not crystallized and therefore has high Li^+^ conductivity (Croce et al., 1998, 2000; D'Epifanio et al., 2004; Zhang et al., 2017).

### Challenges of ASSB in Cycles: Mechanical Integrity for Long-Term Cycles

**Figure 8:**
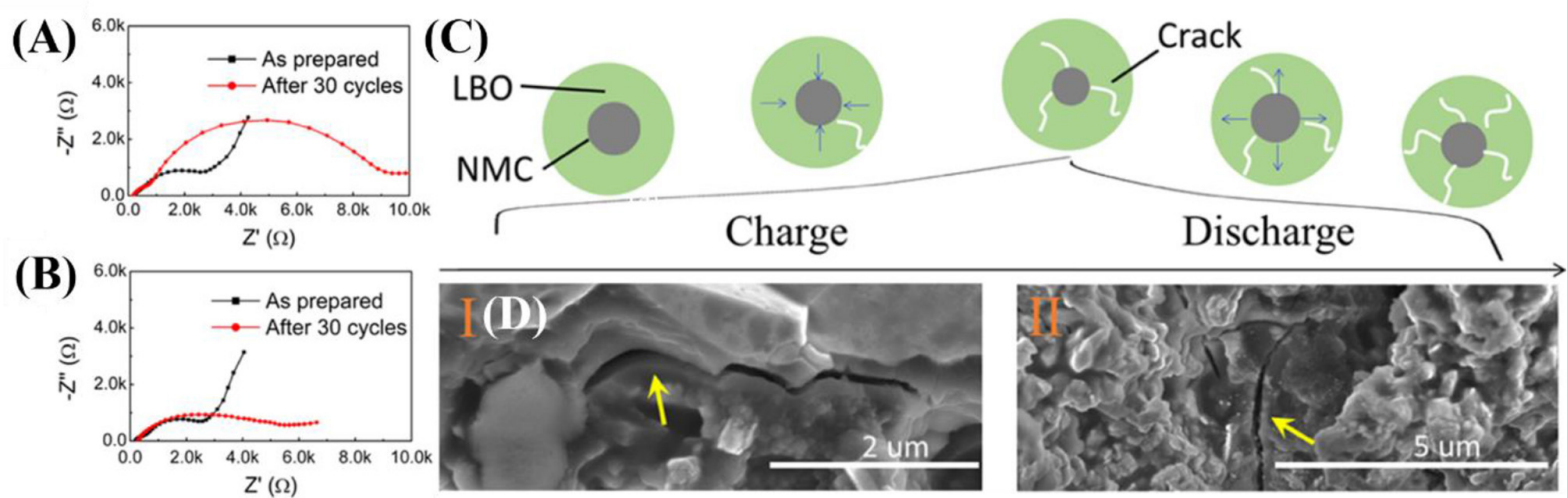
EIS **(A)** large, **(B)** small - NMC after 30 cycles, composite cathode **(C)** schematic of the cathode composite during cycle **(D)** SEM images for composite cathode after 30 cycles [Reproduced from Wang M. et al. (2019) with permission from the American Chemical Society].

For ASSB to be used in practical applications, reliable cycling retention is an essential property. Dense SE and dense cathode structure are preferred in ASSB to improve electrochemical activity by reducing interfacial resistances. The volume change caused by the insertion/extraction of Li for cycles in ASSB therefore severely affects the mechanical integrity of the cell. Loss of mechanical integrity in an ASSB decreases ionic conductivity and electrochemical activity and causes poor cycle stability with reduced energy density. To increase energy capacities in ASSB, LCO has being replaced by high-capacity Ni-rich materials such as LiNi_0.8_Mn_0.1_Co_0.1_O_2_. However, Ni-rich materials undergo much larger volume change than LiCoO_2_ during cycles because they use almost all Li^+^ from the layered structure (Ryu et al., 2018). As a result, large volume change can severely cause mechanical damage, such as cracking, which severely increases in ASSB, which use Ni-rich materials as an active material. Therefore, use of high-capacity cathode materials complicates the task of increasing the capacity retention in ASSBs. Minimizing the particle size in cathode can be one of effective ways to decrease the formation of cracks and thereby reduce the mechanical damage in ASSB (Figure 8) (Wang D. et al., 2019). Furthermore, cathode materials that show negligible volume changes such as cation-disordered materials may be useful to minimize the mechanical damages of ASSB during cycling. Before ASSBs can be applied practically, the cause of mechanical damage due to volume change during cycling must be understood and the problem solved.

## Summary

This review has surveyed the characteristics of currently developed garnet-type LLZO SEs and their interfaces with Li metal and cathode. We have focused on the requirements to achieve high energy density ASSBs with respect to materials' electrical properties, interfacial reactions, and fabrication process. Garnet-type LLZO SEs have high Li^+^ conductivity of ~10^−3^ S·cm^−1^ and high chemical and electrochemical stability against Li metal. Due to these characteristics, they are considered to be suitable to achieve high energy density oxide SEs. However, before ASSB with LLZO SEs can be used in practical applications, several problems must be solved. (1) The poor wetting behavior of LLZO with Li metal should be overcome to achieve low interfacial resistance. (2) The critical current density of LLZO SEs should be understood and improved to enable use of Li metal as an anode without the growth of Li dendrites. (3) The most important challenge is to develop reliable processes to fabricate the interface between cathode and SEs and to construct reliable ASSB with oxide SEs. If these problems can be solved, ASSBs can become competitive with batteries that use liquid electrolytes.

## Author Contributions

BK contributed to the idea. All the authors discussed the results and reviewed the manuscript.

## Conflict of Interest

The authors declare that the research was conducted in the absence of any commercial or financial relationships that could be construed as a potential conflict of interest.
